# Increased Reliance on Carbohydrates for Aerobic Exercise in Highland Andean Leaf-Eared Mice, but Not in Highland Lima Leaf-Eared Mice

**DOI:** 10.3390/metabo11110750

**Published:** 2021-10-29

**Authors:** Marie-Pierre Schippers, Oswaldo Ramirez, Margarita Arana, Grant B. McClelland

**Affiliations:** 1 Department of Biology, McMaster University, 1280 Main Street West, Hamilton, ON L8S 4K1, Canada; schippersm@gmail.com; 2 Unidad Biología Integrativa, Departamento de Ciencias Biológicas y Fisiológicas, Facultad de Ciencias y Filosofía, Universidad Peruana Cayetano Heredia, Lima 15102, Peru; oswaldo.ramirez@upch.pe (O.R.); maria.arana@upch.pe (M.A.)

**Keywords:** altitude, carbohydrates, respirometry, aerobic capacity, fuel use, muscle

## Abstract

Exercise is an important performance trait in mammals and variation in aerobic capacity and/or substrate allocation during submaximal exercise may be important for survival at high altitude. Comparisons between lowland and highland populations is a fruitful approach to understanding the mechanisms for altitude differences in exercise performance. However, it has only been applied in very few highland species. The leaf-eared mice (LEM, genus *Phyllotis*) of South America are a promising taxon to uncover the pervasiveness of hypoxia tolerance mechanisms. Here we use lowland and highland populations of Andean and Lima LEM (*P. andium* and *P. limatus*), acclimated to common laboratory conditions, to determine exercise-induced maximal oxygen consumption ($\dot{V}$O_2_max), and submaximal exercise metabolism. Lowland and highland populations of both species showed no difference in $\dot{V}$O_2_max running in either normoxia or hypoxia. When run at 75% of $\dot{V}$O_2_max, highland Andean LEM had a greater reliance on carbohydrate oxidation to power exercise. In contrast, highland Lima LEM showed no difference in exercise fuel use compared to their lowland counterparts. The higher carbohydrate oxidation seen in highland Andean LEM was not explained by maximal activities of glycolytic enzymes in the gastrocnemius muscle, which were equivalent to lowlanders. This result is consistent with data on highland deer mouse populations and suggests changes in metabolic regulation may explain altitude differences in exercise performance.

## 1. Introduction

Exercise is an important performance trait that can significantly impact fitness and survival in wild mammals [1,2]. The capacity for aerobic exercise depends on the coordination of many physiological traits supporting muscle metabolism by ensuring adequate supply of oxygen and the appropriate metabolic substrates. In studies where capacity for aerobic locomotion has been measured, it has been found to vary with body mass, life-history, and environment stressors (see [3,4,5,6]). Substrate use during submaximal aerobic exercise has been much less studied, but rates of oxidation of carbohydrates and lipids, the two major fuels used by exercising mammals, varies directly with differences in aerobic capacity [4]. That is, a mammal with an elevated aerobic capacity would be expected to use glucose and fatty acids at a higher rate when at the same relative submaximal exercise intensities (as % of maximal oxygen consumption, $\dot{V}$O_2_max) as a more sedentary species. The result is equivalent proportional use of metabolic substrates (as % of total oxygen consumption, $\dot{V}$O_2_) across several taxa of lowland native mammals [4,7]. However, mammals living in extreme environments may deviate from this predictable pattern of fuel use to improve exercise performance. Yet, there is surprisingly few studies examining how exercise metabolism in wild mammals varies across environments.

The low oxygen availability in high altitude environments is known to restrict aerobic exercise performance in lowland native mammals [8,9,10]. For this reason, some species living at high altitude have evolved an elevated running $\dot{V}$O_2_max in hypoxia [3,11,12]. Although exceptions exist [3], suggesting that elevated $\dot{V}$O_2_max in hypoxia may not be a universal trait at high altitude. During submaximal exercise performance in hypoxia could be enhanced by economizing the O_2_ available in the high alpine [13]. Increased carbohydrate use by working muscles has been hypothesized to provide an efficiency advantage in hypoxic environments [13,14] because of a superior ATP yield per O_2_ consumed compared to lipid oxidation [15,16,17]. This hypothesis was first supported by data from two species of leaf-eared mice (LEM) living at high altitude in the Peruvian Andes (*Phyllotis xanthopygus* and *P. andium*), which showed a greater proportional use of carbohydrates to power submaximal exercise than two sister species living at low altitude (*P. limatus* and *P. amicus*), after all mice were acclimated to common low altitude conditions [3]. However, the pervasiveness of this trait in high altitude mammals is unclear.

Intraspecific comparisons of lowland and highland populations have also proven successful to understand how certain traits have evolved at high altitude. Highland populations of humans and North American deer mice (*Peromyscus maniculatus*) have both provided convincing evidence of ongoing selection on several physiological traits related to the oxygen transport pathway [12,18,19,20,21,22,23,24]. Recent research has shown that differences between lowland and highland populations of deer mice living at the same latitude are the result of both fixed genetic differences and evolved phenotypic plasticity [22]. This includes an increased aerobic capacity and greater reliance on carbohydrates to power submaximal exercise [5,12]. Yet, these comparisons may provide limited knowledge on the pervasiveness of specific high altitude physiological traits across highland populations. The leaf-eared mice of South America have been less studied than their North American counterparts (e.g., [25,26]), but species of this Genus have a broad geographical distribution throughout South America [27]. Many of these species also show broad population distributions, including along large altitude clines [28]. Thus, this group holds promise in undercovering if mechanisms of hypoxia tolerance vary across highland taxa.

To explore interpopulation variation in aerobic capacity and submaximal exercise fuel use, we used two species of LEM that exist along a wide altitude cline and compare wild-caught individuals from highland and lowland populations. The Andean LEM (*P. andium*) is abundant at high elevations in the Peruvian Andes but has an isolated lowland population [27]. In contrast, highland populations of Lima LEM (*P. limatus*) may have experienced gene flow with lowland individuals that constrained their ability to fully adapt to high altitude. We acclimated male mice for at least 6 weeks to common laboratory conditions at low altitude. This reduces but does not eliminate the influence of phenotypic plasticity on physiological variables. We investigated exercise induced $\dot{V}$O_2_max, whole-body rates of fuel oxidation at a submaximal exercise intensity, and biochemical phenotypes of skeletal muscle. We tested the hypothesis that, like highland species within the genus *Phyllotis* [3], mice from highland populations will also rely to a greater extent on carbohydrates to power submaximal exercise than their lowland conspecifics. We further predict this difference in fuel use will be reflected in muscle metabolic capacity for carbohydrate oxidation. Our results show that highland populations of both species had similar $\dot{V}$O_2_max than their respective lowland populations. However, when run at the same % of $\dot{V}$O_2_max only the highland Andean LEM relied on carbohydrates to a greater extent than did its lowlander counterpart. Surprisingly, skeletal muscle of highland mice did not show higher maximal activities for enzymes in glycolysis but did have a higher muscle oxidative capacity than lowlanders, which may facilitate physiological functions in hypoxic environments.

## 2. Results

After at least 6 weeks residence in identical laboratory conditions, there was no significant difference in body weight between lowland and highland Andean LEM (Table 1). However, significant differences in mass were seen between lowland and highland populations of Lima LEM. Thus, mass at the time of each measurement was included in our statistical analysis for this species.

### 2.1. Resting Metabolism

Resting metabolic rates ($\dot{V}$O_2_) in the post absorptive state were similar in lowland and highland Andean LEM when determined in either normoxia or hypoxia (Table 1). When exposed to acute hypoxia, mice from the highland population showed an increase in RER to a value significantly higher than lowland mice in the same condition (0.82 ± 0.01 versus 0.77 ± 0.01, for highland and lowland mice; *p* < 0.05). Similarly, resting $\dot{V}$O_2_ did not differ between populations of Lima LEM (Table 2) with lowland and highland mice expending similar amounts of energy in both normoxia and hypoxia. RER was also similar between populations regardless of experimental condition, but lowland Lima LEM showed a significant decline in RER in acute hypoxia from 0.80 ± 0.01 in normoxia to 0.76 ± 0.01 (Table 2).

### 2.2. Maximal Oxygen Consumption and Aerobic Scope

**Figure 1:**
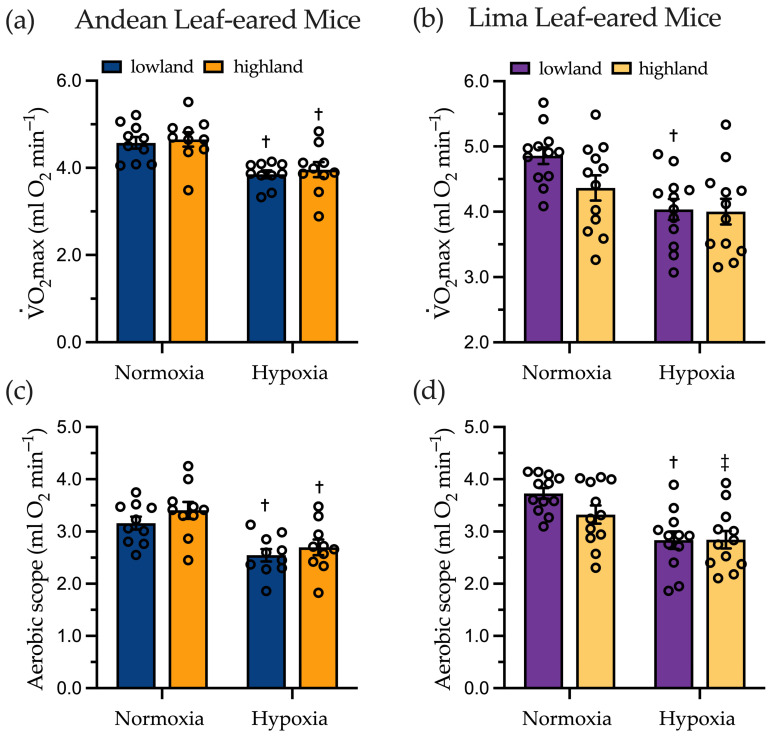
Exercise induced maximal oxygen consumption ($\dot{V}$O_2_max) determined for lowland and highland male (**a**) Andean leaf-eared mice (*Phyllotis andium*) (N = 10 in normoxia and N = 10 in hypoxia) and (**b**) Lima leaf-eared mice (*P. limatus*), in normoxia and hypoxia (N = 12). For Andean leaf-eared mice there was a significant effect of condition (hypoxia vs. normoxia, F = 90.21, *p* < 0.001) but not for population (F = 0.250, *p* = 0.626), nor was there a population × condition interaction (F = 0.031, *p* = 0.863). For Lima leaf-ear mice, there was a significant difference in body mass (F = 49.78, *p* < 0.001) and also a significant effect of condition (F = 23.79, *p* < 0.001) on $\dot{V}$O_2_max. Aerobic scope ($\dot{V}$O_2_max−resting $\dot{V}$O_2_) for (**c**) Andean leaf-ear mice showed a significant effect of condition (F = 38.87, *p* < 0.001), and in (**d**) Lima Leaf-eared mice aerobic scope also showed a significant effect of condition (F = 32.38, *p* < 0.001) and an interaction effect that approached significance (F = 3.97, *p* = 0.053). ^†^ Significantly different than normoxia within a population (*p* < 0.05), ^‡^ *p* < 0.06.

To account for the relationship between exercise intensity relative to maximal aerobic capacity and use of carbohydrates and lipids [4], we determined $\dot{V}$O_2_max while running in normoxia and hypoxia for each mouse. We found for both species that $\dot{V}$O_2_max did not differ between lowland and highland populations. Acute hypoxia led to a significant decrease in $\dot{V}$O_2_max of ~16% in lowland and highland Andean LEM (*p* < 0.001) and ~17% in lowland Lima LEM (*p* < 0.001). Running in hypoxia highland Lima LEM showed ~8% drop in $\dot{V}$O_2_max that was not statistically different from running in normoxia (*p* = 0.263) (Figure 1a,b). Similarly, aerobic scope ($\dot{V}$O_2_max−resting $\dot{V}$O_2_) was not different between lowland and highland populations in each species within an experimental condition. Aerobic scope declined in acute hypoxia in Andean LEM by ~19% in lowland (*p* < 0.001) and ~14% in highland individuals (*p* < 0.001) (Figure 1c). In Lima LEM aerobic scope decline in hypoxia by ~24% for lowland (*p* < 0.001) and ~15% for highland individuals, approaching statistical significance (*p* = 0.058) (Figure 1d).

### 2.3. Submaximal Exercise Metabolism

**Figure 2:**
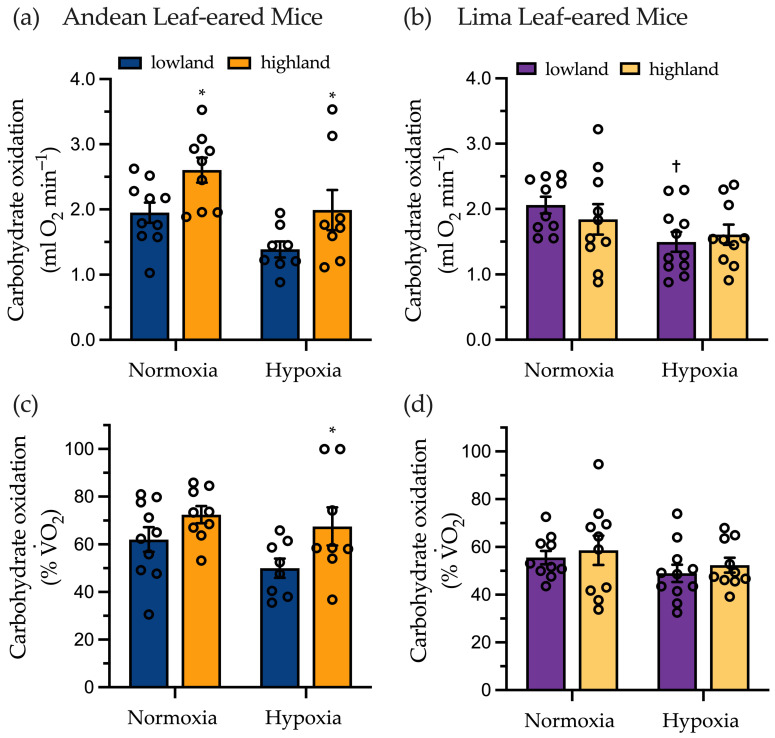
Absolute rates of carbohydrate oxidation during submaximal exercise in lowland and highland male (**a**) Andean leaf-eared mice (*Phyllotis andium*) (N = 10 and N = 9 for lowlanders and highlanders in normoxia, and N = 8 for both populations in hypoxia) and (**b**) Lima leaf-eared mice (*P. limatus*) (N = 10 for both populations in normoxia and N = 11 and N = 10 for lowlanders and highlanders in hypoxia). Lowland Andean leaf-eared mice were running at 75.5 ± 0.9% $\dot{V}$O_2_max in normoxia and 75.9 ± 2.2% $\dot{V}$O_2_max in hypoxia. Exercise intensities for highlanders were 76.1 ± 0.6% and 72.1 ± 1.9% $\dot{V}$O_2_max. Carbohydrate oxidation showed a significant effect of population (F = 9.62, *p* = 0.004) and condition (F = 8.45, *p* = 0.007). Lowland Lima leaf-eared mice were running at 74.3 ± 0.8% $\dot{V}$O_2_max in normoxia and 77.5 ±0.6% $\dot{V}$O_2_max in hypoxia. Exercise intensities for highlanders were 74.2 ± 0.9% and 75.6 ± 0.9% $\dot{V}$O_2_max. In this species mass was significantly different (F = 11.41, *p* = 0.002) at the time of measurement of carbohydrate oxidation which showed a significant effect of condition (F = 13.95, *p* < 0.001). When carbohydrate oxidation was expressed as a % of total $\dot{V}$O_2_ (**c**) Andean leaf-eared mice showed a significant effect of population (F = 6.75, *p* = 0.014). For (**d**) Lima leaf-eared mice there were no significant effects of population or condition. * Significantly different from lowland population with a species. ^†^ Significantly different from normoxia within a population.

Mice were run at a target intensity of 75% $\dot{V}$O_2_max to determine substrate oxidation rates during submaximal exercise. Andean LEM were exercising in normoxia at an intensity of 75.5 ± 0.9% $\dot{V}$O_2_max for lowlanders and 76.1 ± 0.6% $\dot{V}$O_2_max for highlanders. We found that highlanders had higher $\dot{V}$O_2_ and absolute rates of carbohydrate oxidation (+1.33-fold, *p* = 0.047) than lowland Andean LEM (Table 1 and Figure 2a). Overall, the fraction of total $\dot{V}$O_2_ that was used to support carbohydrate oxidation was 1.17-fold higher in the HA mice compared to their LA counterparts (72.4 ± 3.6% versus 62.0 ± 5.2% of total $\dot{V}$O_2_; Figure 2c). However, this increase in relative use of carbohydrates did not reach statistical significance (*p* = 0.162). In contrast, Lima LEM showed no difference in $\dot{V}$O_2_ or absolute substrate use when exercising in normoxia (Table 2 and Figure 2b,c).

Treadmill speed was adjusted for running in hypoxia to account for the decline in $\dot{V}$O_2_max to maintain exercise intensity at 75.9 ± 2.2% $\dot{V}$O_2_max and 72.1 ± 1.9% $\dot{V}$O_2_max for lowland and highland Andean LEM, respectively. Running in hypoxia, highland Andean LEM showed higher RER, higher absolute carbohydrate oxidation and a greater proportional use of carbohydrates (as % total $\dot{V}$O_2_), compared to lowlanders (Table 1, Figure 2a,c). In these conditions, absolute rate of carbohydrates use was 1.43-fold higher (*p* = 0.052), carbohydrates were relied upon to a significantly greater extent (67.5 ± 7.9% versus 50.0 ± 4.0% total $\dot{V}$O_2_, *p* = 0.039) and there was a lower reliance on lipid oxidation (32.4 ± 7.9 versus 49.9 ± 4.0% total $\dot{V}$O_2_) that approached statistical significance (*p* = 0.068), than in lowland Andean LEM (Table 1, Figure 2). In contrast, lowland and highland Lima LEM showed no difference in exercise fuel use when running in normoxia at 74.3 ± 0.8% and 74.2 ± 0.9% $\dot{V}$O_2_max, respectively (Figure 2b). When running in hypoxia at 75.8 ± 1.3%.

$\dot{V}$O_2_max, lowland Lima LEM showed reduced $\dot{V}$O_2_ and rates of carbohydrate oxidation compared to in normoxia. This resulted in no change in the relative contribution of carbohydrate oxidation to total $\dot{V}$O_2_ compared to running in normoxia (55.6 ± 2.8% versus 49.0 ± 3.6% total $\dot{V}$O_2_) (Figure 2d).

### 2.4. Skeletal Muscle Enzyme Activities

**Figure 3:**
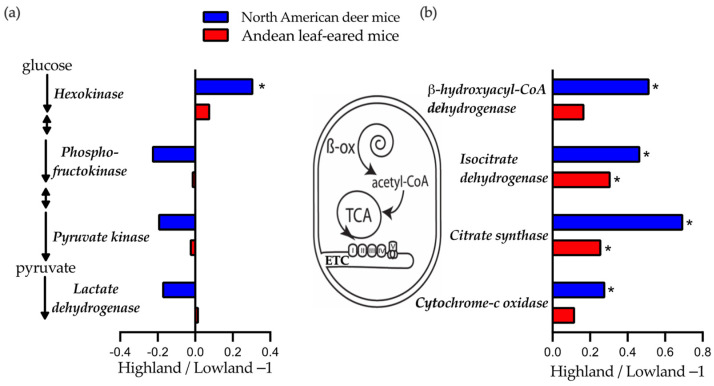
Mean enzyme activities in the gastrocnemius muscle of highland mice relative to lowland mice of the same species (Vmax Highland/Vmax Lowland-1). These enzymes are involved in (**a**) glycolysis and (**b**) mitochondrial ß-oxidation (ß-ox; ß-hydroxyacyl-CoA dehydrogenase), the tricarboxylic acid cycle (TCA; Isocitrate dehydrogenase, citrate synthase) and the electron transport chain (ETC; cytochrome-c oxidase). Data are for wild-caught Andean leaf-ear mice acclimated to common lowland conditions (this study) and North American deer mice (*Peromyscus maniculatus*) born and raised in common lowland conditions and acclimated to hypobaric hypoxia simulating 4300 m altitude [5,12]. In both species mice from highland populations had a greater reliance on carbohydrate oxidation during submaximal exercise. * Significantly different from lowland mice within a species.

To help understand what underlying traits might explain population difference in exercise fuel use for Andean LEM, we examined the metabolic capacities in skeletal muscle at several enzyme steps by determining their apparent Vmax. The maximal activities of enzymes involved in glycolytic, β-oxidation, and aerobic metabolism pathways were measured in the gastrocnemius and thigh muscle (Table 3 and Table A1). In the gastrocnemius there were no significant differences between the lowland and highland Andean LEM in Vmax for the four glycolytic enzymes measured (Table 3, Figure 3a, *p* > 0.05). Nor was the activity of HOAD, an important enzyme involved in the β-oxidation of fatty acids, significantly elevated in the HA population (*p* = 0.07). On the other hand, the activities of the enzymes CS (*p* = 0.012) and IDH (*p* = 0.048) were found to be higher in this muscle of the highland mice (Table 3, Figure 3b), suggesting increased tissue aerobic capacity. This is supported by the significantly higher CS/LDH ratio seen in highland Andean LEM (Table 3). Vmax of glycolytic enzymes in the thigh were also not different between the lowland and highland populations, with the exception in LDH, which was higher in highland mice (Table A1, *p* = 0.013). All other enzymes activities in the thigh were not different between the lowland and highland mice. These data are consistent with those from the only other study examining exercise fuel use and muscle phenotype as a function of resident altitude, where whole animal variation in exercise fuel use is not necessarily reflected in muscle enzyme capacities (Figure 3).

## 3. Discussion

The goal of this study was to determine if mice from highland populations of Andean and Lima LEM modify their exercise metabolism, to a greater reliance on carbohydrates. We found that mice from lowland and highland populations had similar exercise-induced $\dot{V}$O_2_max. Running in hypoxia led to a decrease in $\dot{V}$O_2_max but no population differences were observed in either species. When mice were run at 75% of their individual $\dot{V}$O_2_max, highland Andean LEM showed higher absolute rates of carbohydrate oxidation and a greater reliance on this fuel to power exercise than seen in a lowland population. In contrast, highland Lima LEM showed no difference in fuel use running at 75% $\dot{V}$O_2_max compared to their lowland counterparts, suggesting changes in exercise fuel use may not be a universal adaptation at high altitude. The greater carbohydrate oxidation seen in highland Andean LEM was not correlated with gastrocnemius muscle maximal in vitro activities of glycolytic enzymes, which were equivalent to those in lowlanders. This result is consistent with data from highland deer mice and suggests alternate underlying mechanisms, such as metabolic regulation of key steps in glucose metabolism, explain altitude differences in exercise fuel use.

### 3.1. Aerobic Capacity and Aerobic Scope

A high aerobic capacity in hypoxia can be beneficial for highland native animals, as it can allow for a greater aerobic scope ($\dot{V}$O_2_max−$\dot{V}$O_2_rest) and aerobic reserve ($\dot{V}$O_2_max−field metabolic rate) allowing animals in these environments the capacity to increase aerobic activities. Indeed, wild-caught yellow-rumped LEM from 4500 m, acclimated to lowland condition for 6 weeks, show a higher exercise induced $\dot{V}$O_2_max in hypoxia than two species from low altitude [3]. Highland and lowland native deer mice born and raised in common lab condition show a significant population difference in exercise-induced $\dot{V}$O_2_max in hypoxia with highlander showing a greater aerobic capacity [12]. However, we found no difference in $\dot{V}$O_2_max between lowland and highland populations in either Andean LEM or Lima LEM running in normoxia or hypoxia.

This is consistent with some studies showing highland native humans have similar aerobic capacities to acclimated lowlanders (reviewed in [29]). These data suggest that an elevated aerobic capacity in hypoxia may not be a hallmark of all highland mammals. Aerobic scope was also greater in highland (4500 m) yellow-rumped LEM in hypoxia [3]. However, not all highland LEM species show an increased aerobic capacity or aerobic scope for exercise [3]. This was also the case for highland populations within the two LEM species studied here.

Highland mammals can also maintain high capacities for thermogenesis to remain active in the cold high alpine [30,31]. Cold-induced $\dot{V}$O_2_max can even exceed exercise-induced $\dot{V}$O_2_max in some cases (reviewed in [6]). Thus, cold-induced $\dot{V}$O_2_max may differ between the lowland and highland populations studied here. However, to study variation in exercise substrate use, it is most appropriate to standardize intensity relative to exercise-induced $\dot{V}$O_2_max, as muscle is the main O_2_ consumer during locomotion [32] Standardizing exercise intensity relative to $\dot{V}$O_2_max is also important to account for the relationship between exercise intensity and the mix of substrates used [4].

### 3.2. Substrate Use at Rest and Submaximal Exercise

Our previous work using four species of LEM and a phylogenetic comparative approach, was the first to strongly infer that an increased reliance on carbohydrate oxidation has evolved as a metabolic strategy for submaximal exercise at high altitude [3]. This trait has also been seen in highland deer mice running in hypoxia [5]. This increase in glucose oxidation would provide an O_2_ saving advantage as demonstrated in other animals [17]. The efficient use of O_2_ would undoubtedly be advantageous at high altitude where O_2_ availability is reduced, but carbohydrates represent a small proportion of body energy stores in mammals [6]. So, while there is tantalizing evidence that increasing exercise carbohydrate use is a common trait shared by highland mice, in the fasted state their stores would become depleted in ~20 min of running at 75% $\dot{V}$O_2_max [6]. For this reason, perhaps not all highland mammals rely on this mechanism to enhance exercise performance in hypoxia. Consistent with this idea, highland Lima LEM showed the same absolute and proportional use of carbohydrate oxidation than did lowland mice. These data also highlight the importance of replication to uncover potential variation in exercise metabolism across different highland taxa.

### 3.3. Muscle Enzyme Vmax

An increased reliance on carbohydrate use has now been observed in running highland deer mice and highland Andean LEM [5] (Figure 2). This replication allows us to examine whether these linages share common underlying mechanisms responsible for changes in fuel use. We compared population differences in maximal enzyme activity of the gastrocnemius in Andean LEM to those previously published for deer mice [5,12] (Figure 3). Despite in vivo differences in carbohydrate use, lowland and highland Andean LEM showed similar in vitro Vmax for enzymes involved in glycolysis. These results are consistent with our previous intraspecific comparison of low- and high-altitude species of *Phyllotis* mice [3] and with data comparing lowland and highland deer mice [5,12] (Figure 3). However, highland deer mice show higher activities of the enzyme HK after hypoxia acclimation [5] suggesting an enhanced capacity for muscle glucose uptake [33]. Nevertheless, these data suggest that the increase in whole-body carbohydrate use is likely achieved through metabolic rather than hierarchical regulation [34]. Indeed, in vivo reaction rates likely reflect changes in cellular conditions during contraction but the relative homeostatic levels of metabolic regulators in working muscle makes this difficult to assess [35]. Possibly, covalent activation of key reaction steps during muscle contraction in hypoxia, such as dephosphorylation of pyruvate dehydrogenase [36] may differ in highland species. High activities of mitochondrial enzymes in the gastrocnemius suggest that muscle oxidative capacity is elevated in highland mice (Figure 3). The gastrocnemius muscle of highland Andean LEM showed significantly higher CS/LDH, evidence of a muscle geared towards aerobic metabolism (Table 3). These population differences are much more dramatic in deer mice and likely reflect the significant difference in mitochondrial density and exercise-induced $\dot{V}$O_2_max seen in this species [12,21].

We also examined enzyme activities in the thigh which encompasses several different skeletal muscles, but likely contains more glycolytic muscle fibres than the gastrocnemius. Both populations of Andean LEM showed similar in vitro enzyme activities except for LDH, which was higher in the highland population. An increase anaerobic capacity may be advantageous for burst activity, but predatory pressure is likely similar in both low and high-altitude environments (Ramirez, O., personal observations). As the by-products of anaerobic metabolism can negatively affect cell function through pH imbalances [37], anaerobic glycolysis is thought to be downregulated in lowlander mice exposed to chronic compared to acute hypoxia [36]. In fact, several studies have reported reductions in muscle anaerobic capacity in high altitude mammals, including deer mice [12,38,39].

### 3.4. Phenotypic Plasticity and Interpopulation Phenotypic Differences

The physiology of highland mammals is the result of fixed genetic traits and/or adaptive variation in phenotypic plasticity over developmental and physiological time scales. In our experiments we minimized environmental influences on population differences by acclimating adult mice to common lab conditions at low altitude. The population differences in exercise fuel use are suggestive of a genetically fixed trait in highland Andean LEM. In contrast, highland deer mice born and raised in lab conditions only increased reliance on carbohydrates after hypoxia acclimation. These data suggest that physiological plasticity can be very important to establishing this trait in some but not all species. Results from Lima LEM born and raised in the wild at high altitude but acclimated to low altitude as adults, suggest developmental plasticity alone does not lead to changes in exercise fuel use as adults in this species. Future work on LEM would need to breed mice from different altitudes in common garden conditions and acclimate these mice to simulated high altitude during development and as adults. This has been a successful experimental approach to uncover gene × environment interactions in deer mice (e.g., [40,41,42]). This replication will highlight if different lineages of mice show distinct approaches for adaptation to high altitude.

**Figure A1:**
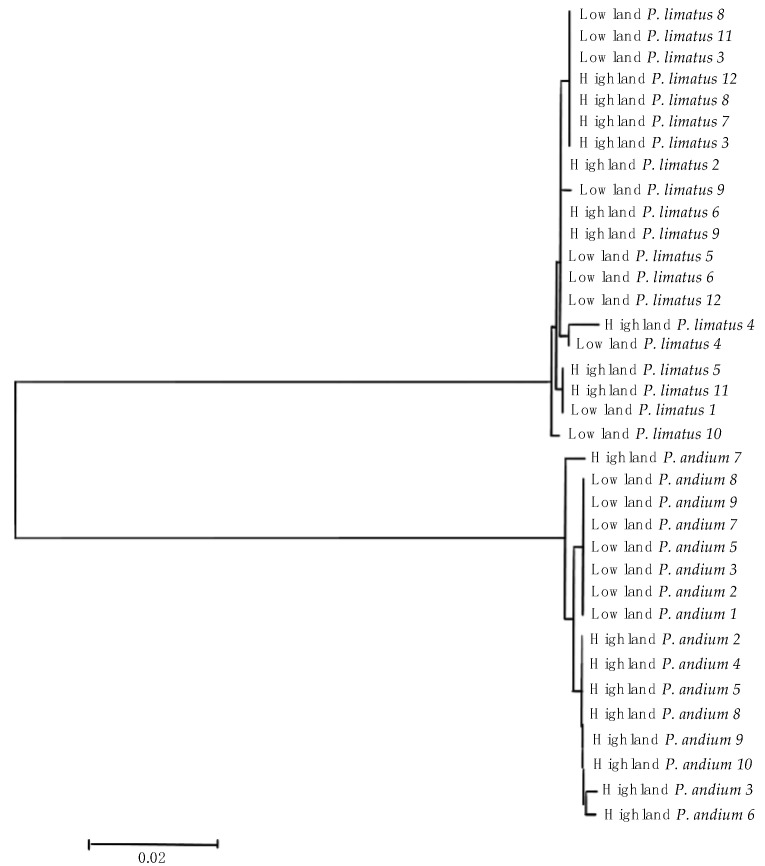
Phylogenetic relationships of individuals from the lowland and highland populations of Andean leaf-eared mice (*Phyllotis andium*) and Lima leaf-eared mice (*P. limatus*). Each individual mouse is labeled with an arbitrary number Tree based on cytochrome *b* gene sequences as previously described [3].

Any physiological change that occurs along an altitude cline must balance gene flow and diversifying variation in changing environments. The influx of lowland genes due to recent or ongoing gene flow in both humans [43] and deer mice [44] could affect the ability of highland populations to fully adapt to high altitude. In contrast, in the Andes of South America population isolation is promoted by the rugged and deeply dissected landscape. The Andean LEM are abundant at elevations ranging from 1000 and 4100 m in the Peruvian Andes, and has an isolated population found close to sea level [27]. The degree of geographic separation suggests there may be low rates of inter-population gene flow (in either direction), and consequently Andean LEM may be well suited to investigate physiological phenotypes in different populations that inhabit distinct altitudinal ranges. In contrast, highland and lowland populations of Lima LEM may have experienced recent or ongoing gene flow, as suggested by shared haplotypes (Figure A1). It seems likely that this gene flow has constrained their ability to fully adapt to high altitude. It is also possible that different highland populations employ distinct mechanisms for effective submaximal exercise in hypoxia [45] that do not rely on changes in substrate use.

### 3.5. Conclusions

These data show that submaximal exercise substrate use varies in a highland population of LEM independent of differences in $\dot{V}$O_2_max from their lowland conspecifics. The increase in reliance on carbohydrates in highland Andean LEM would make more efficient use of O_2_ compared to lipid oxidation, but with the risk of depleting the limited stores available to mammals. This may explain why highland Lima LEM do not alter exercise fuel use compared to lowlanders. It is also possible that this species relies to a greater extent on phenotypic plasticity to express this change in substrate use, similar to highland deer mice [5].

Nevertheless, LEM hold great promise in advancing high altitude research by undercovering common and distinct evolved mechanisms for effective exercise in hypoxia between highland taxa. LEM can also help us understand the physiological limits of altitude distributions in mammals. Populations of LEM exist across some of the widest altitude clines known, with Lima LEM found up to 5070 m and the yellow-rumped LEM to >6700 m [28]. These limits may be set by the limitations to aerobic exercise in face of declining O_2_ availability.

## 4. Materials and Methods

### 4.1. Study Design

We captured male Andean and Lima LEM (*Phyllotis andium* and *Phyllotis limatus*) from two geographically distinct populations. Female mice were not sampled for this study to prevent separating mothers from their pups or including pregnant mice. Lowland Andean LEM were trapped at 300 m a.s.l. in the Reserva Nacional Lomas de Lachay (11°21′582″ S; 77°22′034″ W, N = 10), lowland Lima LEM were trapped at 100 m at Morro Sama (Tacna) (18°01′227″ S; 70°50′013″ W, N = 12), and highland Andean LEM at a site at 4000 m a.s.l. (Marcahuasi, 11°78′928″ S; 76°57′558″ W, N = 10) and Lima LEM at 3700 m (Solabaya) (17°24′59″ S, 70°30′40″ W, N = 12). Mice were genotyped to confirm species identification (Figure A1) as previously [3]. It is important to note that in a recent taxonomic revision of *P. andium*, the two populations used in this study have been reassigned as *P. occidens* sp. nov. [46], but we retain the original species name for consistency with other studies [3]. These populations show high site fidelity. For example, mean travel distances for Andean LEM are in the range of 36 to 50 m over a year or less, approximately their life expectancy in the wild [47,48]. Low altitude and high-altitude populations used in this study are separated by ~185 km and ~150 km for Andean LEM and Lima LEM, respectively. Low and high-altitude sites between species are separated by ~1200 km. Mice were captured using baited Sherman traps and then immediately transported to sea level and kept under identical conditions in the animal facility of the UPCH, in Lima, Peru for a minimum of 6 weeks before experimentation. Animals were housed individually in rodent cages with water and rabbit pellets (Agribrands Purina Peru, Lima) provided ad libitum. Photoperiod and temperature were allowed to fluctuate with outdoor conditions (12–13 h daylight, 18–23 °C).

At least six weeks after arrival at sea level, mice were subjected to the following in vivo experimental measurements under both normoxic (normobaric, 21% O_2_) and hypoxic (normobaric but equivalent to 4000 m, 12% O_2_) conditions: (i) running-induced maximal oxygen consumption ($\dot{V}$O_2_max), then $\dot{V}$O_2_ and carbon dioxide production ($\dot{V}$CO_2_) were measured at (ii) rest and (iii) an exercise intensity equivalent to 75% of individual $\dot{V}$O_2_max, as described previously [3]. To determine $\dot{V}$O_2_max, treadmill speed was increased by 3 m min^−1^ every 2 min starting at 4 m min^−1^. Mice were considered to have reached $\dot{V}$O_2_max if they fulfilled 2 of 3 criteria: (1) $\dot{V}$O_2_ remained unchanged as treadmill speed increased, (2) the mouse was unable to maintain position on the treadmill belt, and (3) respiratory exchange ratio (RER) ≥ 1 [3,5]. Treadmill speed for submaximal intensities were determined from the relationship between $\dot{V}$O_2_ and speed, but treadmill speed was also adjusted in real-time to maintain the targeted relative exercise intensity for a period of 20 min. Data from at least 5 min of recording between the 5th and 20th min of exercise were used to calculate group means. Rates of $\dot{V}$O_2_ and $\dot{V}$CO_2_ in the resting metabolic chamber were recorded for at least 45 min and at least 5 min of the lowest $\dot{V}$O_2_ were used as resting metabolism. These measurements were performed in random order between noon and 7 p.m. following a 6 h fast (except for the $\dot{V}$O_2_max test for which mice were not fasted), and each mouse was never subjected to more than 1 measurement per day. We anaesthetized each mouse by placing them in a small container with an isoflurane-soaked cotton ball before performing a cervical dislocation. Subsequently, muscle samples (gastrocnemius and thigh) were harvested, weighed, immediately crushed with pre-cooled aluminum plates, frozen in liquid nitrogen, and then stored at −80 °C. Data from lowland Andean LEM are compared to previously published data of highland Andean LEM, and data from highland Lima LEM are compared to previously published data for lowland Lima LEM [3].

### 4.2. Respirometry

Rates of $\dot{V}$O_2_ and $\dot{V}$CO_2_ were measured in post-absorptive animals at rest in a 600 mL metabolic chamber or during exercise in a rodent treadmill enclosed in a metabolic chamber (working section of ~800 mL) at room temperature (~21 °C) using a flow-through respirometry system (Sable Systems, Las Vegas, NV, USA) as previously described [3]. Briefly, either outside air, scrubbed free of CO_2_ and H_2_O using Drierite (W. A. Hammond, Xenia, OH, USA), soda lime and Ascarite (Fisher Scientific, Pittsburgh, PA), or 12% O_2_ (balance N_2_), was pushed through the metabolic chambers at 600 mL min^−1^ for rest or 2000 mL min^−1^ for exercise, using a combined pump-mass flow meter. A subsample of excurrent air, dried using pre-baked Drierite [49] was pulled through CO_2_ and O_2_ analyzers at 200 mL min^−1^ (FOXBOX, Sables Systems, Las Vegas, NV, USA). The system was determined to be accurate to within ±1% for RER ($\dot{V}$O_2_/$\dot{V}$CO_2_) by burning a known volume of methanol in the chamber and comparing predicted and experimental values.

### 4.3. Muscle Enzymatic Capacities (Apparent Vmax)

Gastrocnemius and whole thigh muscles in Andean LEM only were powdered using a liquid N_2_-cooled mortar and pestle and homogenized on ice with a glass-on-glass homogenizer for 1 min in 20 volumes of homogenizing buffer consisting of (in mM) 100 potassium phosphate (pH 7.2), 5 ethylenediaminetetraacetic acid (EDTA), and 0.1% Triton X-100. Enzymes were selected to represent key reactions in glycolysis, the TCA cycle, β-oxidation pathways, and as indices of tissue aerobic capacity [50], consistent with previous studies on mice and other rodents [3,5,51]. We determine the maximal activity (apparent Vmax) of hexokinase (HK), phosphofructokinase (PFK), pyruvate kinase (PK), lactate dehydrogenase (LDH), β-hydroxyacyl-CoA dehydrogenase (HOAD), citrate synthase (CS), isocitrate dehydrogenase (IDH), and cytochrome c oxidase (COx). Assays were performed in triplicate and control rates (no substrate added) were determined for each assay using previously published methods for mice [3.5] and measured at 37 °C in a Spectromax Plus 384, 96-well microplate reader (Molecular Devices, Sunnyvale, CA, USA).

### 4.4. Calculations and Statistical Analyses

$\dot{V}$O_2_ and $\dot{V}$CO_2_ were calculated using equation 3b of Withers [52]. Rates of carbohydrate and lipid oxidation were calculated assuming 0.746 L O_2_ g^−1^ for carbohydrates and 2.03 L O_2_ g^−1^ for lipids [53] and that the contribution of protein to overall energy expenditure was assumed to be minimal during exercise in post-absorptive state [54]. Fuel use was also determined relative to total oxygen consumption during exercise as between 0 and 100% $\dot{V}$O_2_. Fuel oxidation rates were not determined at rest because protein oxidation can significantly contribute to overall energy production in this condition. Respiratory and substrate use data were analyzed within each species using a two-way analysis of variance (ANOVA) mixed effect model, or an analysis of covariance (ANCOVA) with mass as a covariate (if significant), using GraphPad Prism version 9.1.2 or in R (https://www.r-project.org/) with population and experimental condition (normoxia or hypoxia) as main effects. Post hoc multiple comparisons used either Tukey or Holm–Sidak tests. Data for muscle enzyme activities in Andean LEM were compared using a t-test. Data are presented as means ± SEM.

## Figures and Tables

**Table 1:** Body mass, oxygen consumption ($\dot{V}$O_2_) and respiratory exchange ratio (RER = $\dot{V}$CO_2_/$\dot{V}$O_2_) in normoxia and hypoxia of lowland and highland male Andean leaf-eared mice while resting and during submaximal exercise at a target intensity of 75% $\dot{V}$O_2_max. Absolute rate of lipid oxidation and relative to total $\dot{V}$O_2_ were determined during exercise.

	Normoxia		Hypoxia	
	Lowland	Highland	Lowland	Highland
Mass (g)	43.5 ± 1.9	45.7 ± 3.3	43.3 ± 2.1	46.5 ± 3.4
Resting				
$\dot{V}$O_2_ (mL min^−1^)	1.41 ± 0.12	1.25 ± 0.09	1.31 ± 0.09	1.26 ± 0.05
RER	0.788 ± 0.012	0.787 ± 0.010	0.772 ± 0.008	0.821 ± 0.013 *
Submaximal exercise				
$\dot{V}$O_2_ (mL min^−1^)	3.17 ± 0.09	3.57 ± 0.13 *	2.79 ± 0.15 ^†^	2.89 ± 0.14 ^†^
RER	0.888 ± 0.015	0.919 ± 0.010	0.853 ± 0.012	0.921 ± 0.033 *
Lipid oxidation (mL min^−1^)	1.22 ± 0.19	0.96 ± 0.12	1.44 ± 0.13	0.90 ± 0.23
Lipid oxidation (% $\dot{V}$O_2_)	37.9 ± 5.2	27.5 ± 3.6	49.9 ± 4.0	32.4 ± 7.9 ^‡^

*n* = 10 for all resting data except for *n* = 9 for highlanders in hypoxia. *n* = 10 for lowander and *n* = 9 for highlander submaximal exercise data in normoxia. In hypoxia *n* = 8 for both lowlanders and highlander. * Significantly different from lowland mice in the same condition, ^†^ significantly different than normoxia within a population, ^‡^ Different from lowland mice in the same condition at *p* < 0.07.

**Table 2:** Body mass, oxygen consumption ($\dot{V}$O_2_) and respiratory exchange ratio (RER = $\dot{V}$CO_2_/$\dot{V}$O_2_) of lowland and highland male Lima leaf-eared mice while resting and during submaximal exercise at a target intensity of 75% $\dot{V}$O_2_max in normoxia and hypoxia. Absolute rate of lipid oxidation and relative to total $\dot{V}$O_2_ were determined during exercise.

	Normoxia		Hypoxia	
	Lowland	Highland	Lowland	Highland
Mass (g)	45.6 ± 1.9	39.7 ± 2.3	46.2 ± 2.0	40.8 ± 2.3
Resting				
$\dot{V}$O_2_ (mL min^−1^)	1.13 ± 0.07	1.04 ± 0.07	1.20 ± 0.04	1.16 ± 0.06
RER	0.795 ± 0.008	0.776 ± 0.007	0.763 ± 0.008 ^†^	0.761 ± 0.006
Submaximal exercise				
$\dot{V}$O_2_ (mL min^−1^)	3.70 ± 0.13	3.17 ± 0.09	3.01 ± 0.12 ^†^	3.01 ± 0.14
RER	0.869 ± 0.008	0.878 ± 0.018	0.850 ± 0.011	0.860 ± 0.009
Lipid oxidation (mL min^−1^)	1.64 ± 0.11	1.25 ± 0.19	1.51 ± 0.10	1.40 ± 0.07
Lipid oxidation (% $\dot{V}$O_2_)	44.3 ± 2.8	41.3 ± 6.1	50.9 ± 3.6	47.5 ± 3.1

*n* = 12 and *n* = 11 for resting data from lowland and highland mice, respecively. *n* = 10 for submaixmal exercise data in normoxia. *n* = 11 and *n* = 10 for exericse data in hypoxia for lowlanders and highlanders, respectively. ^†^ significantly different than normoxia within a population.

**Table 3:** Apparent Vmax (in µmoles min^−1^ g^−1^ w.w.) in the gastrocnemius muscle of lowland and highland Andean Leaf-eared mice for hexokinase (HK), phosphofructokinase (PFK), pyruvate kinase (PK), lactate dehydrogenase (LDH), ß-hydroxyacyl-CoA dehydrogenase (HOAD), citate synthase (CS), isocitrate dehydrogenase (IDH), and cytochrome-c oxidase (COX).

-		Lowland	Highland
Glycolytic	HK	1.85 ± 0.13	2.00 ± 0.14
	PFK	42.8 ± 2.8	41.8 ± 2.9
	PK	385.1 ± 22.3	371.9 ± 15.6
	LDH	756.5 ± 33.9	775.3 ± 41.8
ß-oxidation	HOAD	19.9 ± 1.0	22.3 ± 1.5
Aerobic metabolism	CS	27.4 ± 2.0	35.9 ± 2.3 *
	IDH	14.6 ± 1.1	18.4 ± 1.4 *
	COX	11.8 ± 1.9	13.8 ± 2.2
	CS/LDH	0.037 ± 0.003	0.047 ± 0.003 *
	PK/LDH	0.51 ± 0.02	0.49 ± 0.03

* Significantly different than lowland mice. *n* = 10.

## Data Availability

Data is contained within the article.

## Appendix A

**Table A1:** Apparent Vmax (in µmoles min^−1^ g^−1^ w.w.) in the thigh muscle of lowland and highland Andean Leaf-eared mice for hexokinase (HK), phosphofructokinase (PFK), pyruvate kinase (PK), lactate dehydrogenase (LDH), ß-hydroxyacyl-CoA dehydrogenase (HOAD), citrate synthase (CS), and cytochrome-c oxidase (COX).

-	Enzyme	Lowland	Highland
Glycolytic	HK	1.97 ± 0.21	2.25 ± 0.13
	PFK	35.6 ± 3.9	33.2 ± 3.2
	PK	361.1 ± 25.2	413.4 ± 25.4
	LDH	523.3 ± 12.9	557.9 ± 21.3 *
ß-oxidation	HOAD	15.6 ± 2.0	24.5 ± 1.0
Aerobic metabolism	CS	22.6 ± 1.2	24.5 ± 1.4
	COX	12.5 ± 0.9	13.3 ± 1.0

* Significantly different than lowland mice. *n* = 8 for lowlanders and *n* = 10 for highlanders.
